# A Sheathed Spike Gene, *TaWUS-like* Inhibits Stem Elongation in Common Wheat by Regulating Hormone Levels

**DOI:** 10.3390/ijms222011210

**Published:** 2021-10-18

**Authors:** Xuemei Si, Wanxin Wang, Ke Wang, Yunchuan Liu, Jiangping Bai, Yaxiong Meng, Xueyong Zhang, Hongxia Liu

**Affiliations:** 1 Gansu Key Lab of Crop Improvement and Germplasm Enhancement, College of Agronomy, Gansu Agricultural University, Lanzhou 730070, China; sxm1690319267@163.com (X.S.); baijp@gsau.edu.cn (J.B.); 2 Key Laboratory of Crop Gene Resources and Germplasm Enhancement, Institute of Crop Sciences, Chinese Academy of Agricultural Sciences, Beijing 100081, China; aboluoxinxinzi@163.com (W.W.); wangke03@caas.cn (K.W.); liuyunchuan1991@163.com (Y.L.); zhangxueyong@caas.cn (X.Z.)

**Keywords:** sheathed spike, wheat, GA pathway, BR pathway, plant architecture, yield

## Abstract

The elongation and development of wheat (*Triticum aestivum* L.) stem play an important role in plant architecture. The shortened stem would result in a sheathed spike and a low yield in crops. Unraveling the molecular mechanisms underlying a sheathed spike would be beneficial for plant architecture and yield improvement. We identified a novel gene, *TaWUS-like* (*WUSCHEL*-*related homeobox*-*like*), which regulated sheathed spike and plant architecture in wheat. The plant height of overexpression transgenic lines was significantly decreased and the spike was not completely elongated and enclosed in flag leaf sheaths. Moreover, the increase in tiller angle resulted in loose plant architecture and lower yield. The statistical and cytological analysis demonstrated that the length of the uppermost and secondary internode was significantly shortened, especially the uppermost internode which was only half the length of the wild-type. The size of parenchyma cells was obviously reduced and cell length on the longitudinal section was elongated insufficiently compared with wild-type. The analysis of hormone content showed that there was a lack of gibberellin A 3 (GA_3_) in internodes but a higher brassinosteroid (BR) content. *TaWUS-like* may inhibit the synthesis of GA_3_ and/or BR, thus affecting the function of signal transduction of these hormones, which further caused stem shortening and plant dwarfing in wheat.

## 1. Introduction

Plant architecture is a key agronomical factor determining crop yield [1]. The proper tiller number, appropriate height and leaf angle are the basis for ensuring effective lodging resistance, normal pollination and high yield of close planting for wheat. As an important food crop, with the rapid expansion of large population and the shrinking of cultivated land areas, the demand for wheat production will be greatly increased in the future. Therefore, it is an urgent and essential task for wheat breeding to understand and improve wheat yield by the engineering of plant architecture [2,3].

The elongation and development of the uppermost internode play an important role in improving plant architecture, which is regulated not only by genetic factors but also by levels of hormones such as gibberellic acid (GA), auxin (IAA), brassinosteroid (BR) and ethylene (ETHY), etc. Among them, GA is the most important hormone [4]. In rice, the use of hybrid rice has significantly increased rice production. However, the sheathed panicle was a serious and widespread phenomenon in rice hybrid sterile lines which was manifested as the shortened stem and sheathed panicle with varying degrees, causing 30–60% of panicles to fail to spread normally from flag leaf sheath [5], which in turn results in a great reduction of rice quality and yield of hybrids. Studies have shown that this phenomenon is mainly due to the lack of GA content in the stem of hybrid rice [6,7]. In rice, the sheathed panicle was relieved by exogenous spraying GA in the leaves. However, the detailed molecular mechanisms of the sheathed panicle are still unclear.

The trait of sheathed panicle caused by the shortening of the uppermost internode was a typical quantitative genetic trait, which was controlled by both major and minor genes. At present, thirteen QTLs controlling the elongation of the uppermost internodes were identified and eleven mutants with sheathed panicles were obtained through natural variation [8], physical and chemical mutagenesis and transgenic technology. Six mutants with shortened or sheathed panicles (http:www.shigen.nig.ac.jprice/rgn/vol12/pan.htm1, accessed on 1 May 2011) were reported on the Shigen website [9]. These mutants could be classified into full-sheathed or half-sheathed panicles according to the wrapping degree of the sheathed panicle. GA is the main hormone regulating cell elongation of the uppermost internode. Oikawa et al. demonstrated that *Sd1* mutations significantly inhibited GA synthesis in internodes and resulted in retardation of cell elongation and dwarfing of rice [10]. The apparent elongation of the stem in the *eui* mutant was also due to the excessive accumulation of GA_4_ at stem internode in rice. Unfortunately, these studies did not pay much attention to the traits of the sheathed panicle. Zhu et al. cloned and reported two sheathed panicle mutants *sui1-1* and *sui1-2* that showed half-sheathed or full-sheathed panicles, respectively. These two genes are located on rice chromosome 1 and encode a phosphatidylserine synthetase [11]. *SUI1*-RNA interfering lines showed retardation development of the uppermost internode, which is related to insufficient elongation of longitudinal cells of the stem. However, the detailed mechanism of sheathed panicle is still unclear. A *sui2* mutant which was obtained from the tissue culture offspring of *japonica* rice Kitaake with varied half-sheathed or full-sheathed panicles was located on the long arm of chromosome 4 and encoded a cytochrome P450 protein which involved in BR synthesis. Sun et al. found that this gene was allelic to *D11* [5]. Although few rice mutants with sheathed panicles were reported, the underlying molecular mechanisms are still largely unknown, especially in other cereals, such as wheat and maize.

In this study, we cloned and reported one WUSCHEL-related homeobox-like family member which plays a role in the regulation of stem elongation in wheat. When this gene was overexpressed in kenong199(KN199), it caused an obvious altered sheathed spike with a full- or half-wrapped spike by the flag leaf sheath, resulting in a significant decrease in wheat yield. In order to learn the molecular mechanism of the sheathed trait of wheat spike *TaWUS*-*like*, the sheathed trait of the spike, histomorphology of stem internode cells, and the contents of endogenous BR and GA in stem internode cells and flag leaves were observed and determined in both *TaWUS-like* overexpressed material and WT KN199. Transcriptome analysis was performed to investigate the effects of *TaWUS*-*like* overexpression (OE) on GA and BR pathways and cell division-related gene expressions in the grains. To our knowledge, this is the first report of WUSCHEL-related homeobox-like gene-regulating stem development in wheat. 

## 2. Results

### 2.1. Sheathed Spike Phenotype of TaWUS-like-OE Lines

**Figure 1:**
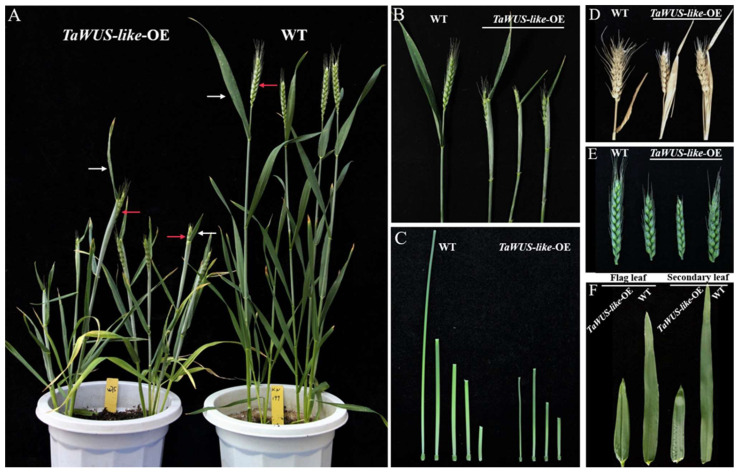
Phenotypes of *TaWUS-like-*OE lines and the WT in wheat at heading stage: (**A**) Lines or plants. The white and red arrows indicate the flag leaf (OE/WT) and the spike (WT)/sheathed spike (OE), respectively. (**B**) Sheathed spike. (**C**) Length of internodes, including uppermost, secondary, third, fourth, fifth from left to right). (**D**) Sheathed spike at mature stage. (**E**) Spike length. (**F**) Flag and secondary leaf.

In this study, a *WUSCHEL*-*related homeobox-like* gene, *TaWUS-like*, was found to result in the sheathed spike when it overexpressed in common wheat cultivar KN199. As shown in Figure 1, the *TaWUS-like*-OE lines showed sheathed spikes that were wrapped full- or half- by the flag leaf sheath (Figure 1A,B) at the heading stage. This phenotype could also be found at the filling stage of wheat, even after harvest, the spike also could not be completely elongated from the leaf sheath (Figure 1D). Moreover, the spike length (Figure 1E) and the plant height of *TaWUS-like*-OE lines decreased obviously, while the tiller angle was enlarged significantly (Figure 1A). Compared with WT, the plant height of *TaWUS*-*like*-OE lines was reduced by nearly two times, and the length of the flag leaf and the second leaf were shortened by about half (Figure 1F). In addition, the spike number and wheat yield were reduced significantly, indicating the vegetative growth of wheat was significantly inhibited by *TaWUS-like*.

In this study, the length and proportion of each internode of *TaWUS-like*-OE lines and WT were evaluated. The results showed that the length of the uppermost and second internode of *TaWUS-like*-OE lines was significantly shorter than those of WT, and the difference was significant (*p* < 0.01). Especially the length of the uppermost internode was only 50% that of WT (Figure 1C); The third and fourth internodes under spike were also slightly shortened in *TaWUS-like*-OE lines compared with WT. Some *TaWUS-like*-OE lines only have four internodes (Figure 1C). However, the length of the last internode of the *TaWUS-like* OE lines was significantly increased compared with that of the WT (*p* < 0.05). Based on the classification of internode shortening types in rice [12], the internode shortening type caused by the *TaWUS-like* gene was defined as “nl” type, that is, the uppermost internode decreased significantly while the last internode was relatively longer. Therefore, the phenomenon of the sheathed spike in wheat by this gene is related to a significant shortening of the internode.

### 2.2. Morphology of Internode of WUS-like-OE Plants at Heading Stage

**Figure 2:**
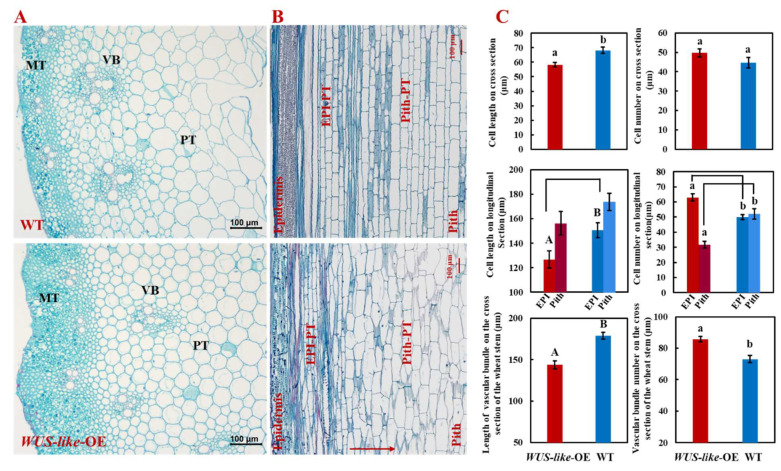
Morphology of the internode of *TaWUS-like*-OE lines and WT at heading stage. (**A**) Cross-section of the uppermost internode (MT: mechanical tissue; PT: parenchyma; VB: vascular bundle); (**B**) Longitudinal section of the uppermost internode (Epi: epidermis; Pith: pith; EPI-PT and Pith-PT indicate the parenchyma cell nearest to epidermis and mid medulla, respectively); (**C**) Data for cell number, size and vascular bundle tissues. Uppercase and lowercase letters indicate *p* < 0.01 and *p* < 0.05, respectively.

To further explore the mechanisms of plant dwarfing and sheathed spike, the cell number and cell size on the cross and longitudinal sections of the uppermost and second internode of *TaWUS-like*-OE lines and WT were evaluated. The results showed that the parenchyma cell size of *TaWUS-like*-OE lines was decreased and the radial diameter of cells per unit area on the cross section was significantly shortened. However, compared with WT, the number of *TaWUS-like*-OE cells was increased (Figure 2A,C). On the longitudinal section, the length of cells of *TaWUS-like*-OE lines was significantly reduced by 15.9%, and the mid-pith region cells were shortened by 10.0% compared with WT. Moreover, the cell shape in the mid-pith region was also changed to irregular related to a regular rectangle in WT (Figure 2B). Moreover, the linear arrangement for the longitudinal cells was also disordered and not as neat as that in the WT (Figure 2B,C). In addition, it was observed that the mechanical tissue of the stem in *TaWUS*-*like*-OE lines was significantly thickened, nearly twice that of the WT. The number, size and deposition direction of the vascular bundles also significantly changed (Figure 2A,C). Taken together, the above results indicated that *TaWUS-like* not only affected the division and number of stem cells but also impacted the elongation of cells on longitudinal. The reduction of parenchyma cell size and insufficient longitudinal elongation of internodes under spike were the main reasons for the appearance of sheathed spikes and dwarf in OE plants.

### 2.3. Changes of Endogenous GA and BR Levels in Flag Leaf and Internodes

**Figure 3:**
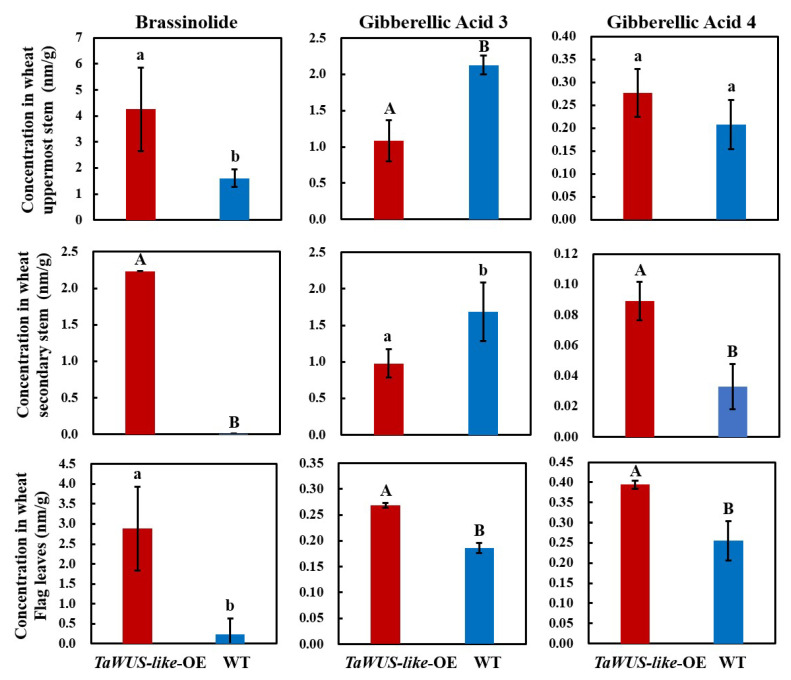
Hormone content determination in the internodes and flag leaf of *TaWUS-like*-OE lines and the WT. Uppercase and lowercase letters indicate *p* < 0.01 and *p* < 0.05, respectively.

Plant dwarfing and internode elongation were usually associated with the changes in endogenous hormone levels in plants. To understand the mechanism of dwarfing and elongation of the stem, the changes of hormone contents in the uppermost and secondary internode and flag leaf were measured. Results showed that the levels of GA and BR in the internodes were significantly changed. Compared with WT, the contents of BR and GA_4_ in the *TaWUS-like*-OE lines were increased in the uppermost, secondary internode and flag leaves. In the flag leaves, the content of BR and GA_4_ was 12.3 and 1.5 times that of WT, respectively. Moreover, the contents of BR and GA_4_ in the uppermost internode were increased about 2.6 (*p* < 0.05) and 1.3 times compared with that of WT, respectively. Moreover, in the secondary internode, the content of BR and GA_4_ was significantly increased 2.2 (*p* < 0.05) and 2.7 (*p* < 0.01) times than that of WT (Figure 3). Meanwhile, the GA_3_ content in flag leaves was increased by 1.44-fold, and the GA_3_ content in the uppermost and secondary internode was reduced by 2-fold (*p* < 0.01) and 1.7-fold (*p* < 0.05) compared *TaWUS-like*-OE lines with WT, respectively, suggesting that different hormones might play different roles during stem elongation. In addition, we found the overall content of GA_4_ was much lower than that of GA_3_ in internode tissues. For example, the content of GA_4_ in the uppermost internode of WT was about 10 times lower than that of GA_3_, while the content of GA_4_ in the secondary internode was about 6 times and 8 times lower than that in the uppermost internode and flag leaf, respectively. In the *TaWUS*-*like*-OE lines, the content of GA_4_ in the uppermost internode was 3.9 times lower than that of GA_3_, and the content of GA_4_ in the secondary internode was about 3 times and 4 times lower than that in the uppermost internode and the flag leaf, respectively. Therefore, the change of GA_3_ content in the internodes may be more important than GA_4_ content in the regulation of internode elongation. The deficiency of GA_3_ and the increase in BR hormone content in the internodes and their balance may be the main causes of internode shortening and plant dwarfing.

### 2.4. TaWUS-like Affected the Expression of GA, BR Pathway and Cell Division Related Genes in Grains

**Figure 4:**
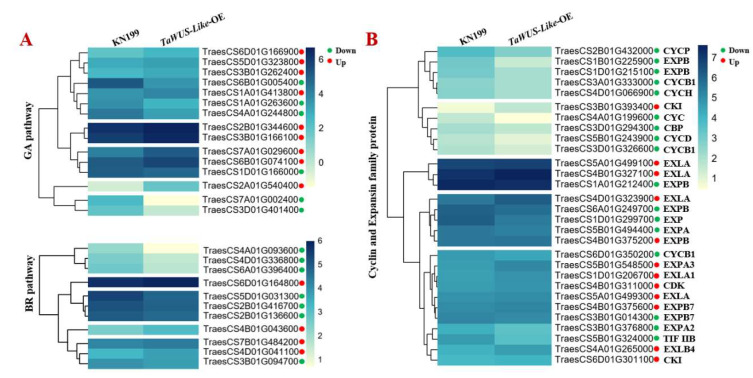
Expression levels of the downstream GA-, BR- (**A**) and cell division-related genes (**B**) regulated by *TaWUS-like-*OE. Red and green circles indicate the expression of the genes was changed upward and downward, respectively.

In order to further investigate the mechanism of a sheathed spike by *TaWUS-like*, the expression nature of GA-, BR- and cell division-related genes was checked using RNA-Seq with 10 days grains after anthesis of *TaWUS-like-*OE and WT. The results showed that the expression levels of 15 genes related to GA synthesis and signal transduction were significantly changed (log2 ≥ 2). Among them, the expression levels of GA synthesis related genes were significantly reduced, such as *GA20ox* (*Traes1A01G263600* and *Traes3D01G401400*), which encoded the key enzymes of the GA synthetic pathway, and terpene synthetase gene (*TraesCS7A01G002400*) was decreased about 2.1, 4.2, and 14.5 times as compared with WT (Figure 4A), respectively. On the other hand, the expression of *GA2ox* (*TraesCS3B01G166100*) and cytochrome P450 gene (*TraesCS6D01G166900*, *TraesCS6B01G074100* and *TraesCS5D01G323800*) which related to GA inactivation, and the expression of *DELLA* (*TraesCS1A01G413800* and *TraesCS3B01G262400*), the important negative regulators of GA signal transduction pathway, were significantly increased with 1.4, 1.6, 1.5, 1.3, 1.4 and 1.4 times higher than that of WT, respectively. Furthermore, it was found that the expression levels of GA signal transduction genes, *TPR* (*TraesCS6B01G005400* and *TraesCS4A01G244800*) were decreased about 3.3 times (*p* < 0.01) and 2.2 times (*p* < 0.01) compared with WT. The results indicated that *TaWUS*-*like* may inhibit the synthesis and function of GA, which was consistent with the phenotype of dwarfing plants with a lower GA content in internodes.

In addition, the expression levels of genes involved in the BR pathway in which five genes related to synthesis and three related to signal transduction were also changed significantly (log2 ≥ 2). The two genes in the BR signal pathway were significantly changed, serine/threonine-protein phosphatase (*TraesCS6A01G396400* and *raesCS4A01G093600*), with decreased 2.1 and 2.8 times compared with WT, respectively. The expression levels of *det* (*TraesCS4B01G043600* and *TraesCS4D01G041100*), which act as the key genes in the synthesis pathway, were significantly increased about 1.5 times and 1.3 times compared with WT. The expression level of *Dim* (*TraesCS7B01G484200*) and cytochrome oxidase genes (*TraesCS6D01G164800*) were increased nearly 1.2 times. The changes in these genes’ activities may be the main reason for the increase in BR content in stems. Moreover, it was found that the gene expression levels of *SMT* (*TraesCS4D01G336800*) and *HYD* (*TraesCS3B01G094700*) were related to the BR synthesis pathway and were significantly inhibited about 2 times and 1.2 times, respectively, compared with WT. These results demonstrated that the regulation of the BR synthesis pathway was refined, in addition, the genes related to the BR pathway have indeed participated in the regulation of stem development. 

As in previous reports, the division and elongation of plant cells are closely related to the function of *Cyclins* and *Expansins* in plants [13]. Here, the expression levels of *Cyclins* and *Expansins* were significantly reduced in *TaWUS-like*-OE lines. Among the 12 altered cell cycle-related genes, the expression of eight cyclin proteins and one initial elongation factor (*TraesCS5B01G324000*) genes were significantly decreased. For example, the gene activities of *TraesCS4A01G199600*, *TraesCS2B01G0432000* and *TraesCS5B01G243900* were reduced by 2.2, 1.9 and 1.8 times, respectively. Two cyclin kinase inhibitors *CKI* gene (*TraesCS6D01G301100* and *TraesCS3B01G393400*), were increased about 1.1 times and 2 times compared with the WT. The activity of the *CDK* gene (*TraesCS4B01G311000*) was increased (about 1.2 times that of the WT). Among the 17 altered expansins family genes, the activities of expansin A and B family genes *EXPA/B* were obviously inhibited, while the gene activity of the expansin A-like family mostly showed an upward trend. Such as *TraesCS1B01G225900* and *TraesCS1D01G215100* of EXPB family and *TraesCS3B01G376800* of EXPA family genes, which were inhibited about 3.1, 1.9 and 1.7 times, respectively, compared with the WT, while the activities of *TraesCS4B01G327100*, *TraesCS4D01G323900* and *TraesCS1D01G206700* of EXLA family genes were increased about 1.3, 1.5 and 1.3 times respectively compared with the WT. These results verified that cell division and elongation were affected by *TaWUS-like*.

## 3. Discussion

The occurrence of the sheathed panicle in rice was mostly related to the insufficient elongation of internodes. Specifically, the longitudinal length of the cell in the longitudinal section was shortened [14]. TaKeda et al. have classified the shortened stem types into five categories based on the proportional ratio of the length of each stem internodes to the whole plant height in rice [12]. Among them, the “sh” type was that the shortening occurs specifically at the uppermost internode. In this situation, the uppermost internode almost has no elongation, and the panicle was sheathed by the flag leaf completely. However, the “nl” type was that the elongation of the uppermost internode was shortened, and the basal internode was elongated. According to the phenotype of sheathed spike caused by *TaWUS-like*, we defined the shortening type as “nl”, which is characterized by an obvious shortening of the uppermost internode and slight elongation of the basal internode. Although the last internode could completely disappear in some *TaWUS-like*-OE lines (Figure 1C), the length of the base internode was always longer than that of WT (*p* < 0.05), indicating the occurrence of a sheathed spike in wheat may also be related to the insufficient elongation of stem cells. In rice, researchers have found various shortening types of internodes [14,15,16,17]. Wang et al. obtained *sui(t)* mutant from JinHui 10 of the *indica* restorer line using EMS mutation showing an extremely shortened uppermost internode with the rice panicle full-wrapped in the leaf sheath, while the other internodes were not changed [15]. Another specific gene *esp2*, mapped on rice chromosome 1, was also showed to control the development of rice uppermost internode. The uppermost internode of the monogenic recessive mutant was almost completely degenerated, and the panicle was completely sheathed by the flag leaf, while the length of the other internodes did not change significantly [14]. In the following research, it was proved that *sui1-1*, *sui1-2* and *esp2* were actually the allelic mutations of each other, encoding a phosphatidylserine synthetase, and the mutation type belongs to “sh” type [16]. Meanwhile, in the rice full-wrapped mutant *fsp*, the shortening occurred from the first internode to the last internodes. However, the proportion of shortened internodes to mutant height is the same as those in WT, thus, mutation belongs to “dn” type [17]. Different genes in different crops caused different shortening phenotypes of the stem may indicate a complex and different network regulating the stem development. Therefore, we suggest that some mechanisms regulating internodes elongation are relatively conserved among different gramineous crops. 

GA and BR are important hormone regulators affecting crop architecture and yield [18]. It was reported that the development of rice uppermost internodes is mainly regulated by these two hormones [5,11,19]. In this experiment, GA and BR contents in flag leaves and internodes of wheat at the heading stage were evaluated. Results showed that the GA_3_ content in the uppermost and secondary internodes of *TaWUS-lik*e-OE lines decreased about 2 times (*p* < 0.01) and 1.7 times (*p* < 0.05) compared with that of WT, respectively, while the BR content increased about 2.3 times (*p* < 0.01) and 2.2 times (*p* < 0.05), respectively. These results indicated that the dwarfing of *TaWUS-lik*e-OE lines and the shortening of internodes may be closely related to the decrease in GA content and the increase in BR level, which is consistent with the research in rice. The reasons for those phenotypes are that the genes regulating the changes of internodes are mostly related to GA synthesis [20,21], catabolism [22] and signal transduction [11,23]. Thus, this may confirm that the mechanism of GA regulating internodes elongation is relatively conserved in different gramineous crops.

In rice, it was also found that the elongation of internodes was regulated by BR [5,24]. As reported by Sun et al., *SUI2* encodes a cytochrome oxidase. The dominant mutation of this single gene can cause a significant shortening of the uppermost internode in rice, which is closely related to the effect of *sui2* on the expression of BR signaling-related genes and the insufficient elongation of the longitudinal cells in stem [5]. Yamamuro et al. have identified a rice dwarf mutant *d61* which was insensitive to BR. The BR level in mutant *d61* was higher than that of the WT, meanwhile, the longitudinal elongation of cells was reduced, and the arrangement of microtubules was distorted [24]. In our study, we found the BR content in the internodes was increased, and the longitudinal elongation of stem cells in the pith region was significantly reduced in *TaWUS-lik*e-OE lines. In addition, the shapes of about 80% of the parenchyma cells in the pith region changed from a regular rectangle to an irregular shape, and the linear arrangement was distorted. Based on the results of previous studies and our current study, we believed that BR was involved in the regulation of the formation and normal elongation of intercalary meristem; however, we speculated that BR may affect the formation of internodes earlier than GA. Hence, the disorder of cell microtubule arrangement was observed in the stem tissues with obvious changes of BR content, while normal phenotype was found for cell shape or arrangement in the stem tissues with only significant changes of GA. Therefore, we believe that the shortening of internodes of wheat is related to the obvious inhibition of cell division and longitudinal elongation by *TaWUS-like*, while GA and BR together are involved in the regulation of genes expression of stem development, which is different from the previous reports that GA or BR alone regulates internode elongation [5,20,25].

Changes in hormone levels are mostly related to the changes in genes expression involved in hormone synthesis or signal transduction. In order to learn the expression characterization of GA- and BR-related genes and understand the molecular mechanism of sheathed spike genes, we analyzed the downstream regulatory genes of *TaWUS-like*. Results showed that the expression levels of key genes related to GA synthesis and signal transduction were significantly inhibited, while the activities of the inactivation-related genes were significantly increased. The gene activities of serine/threonine protein phosphatase related to the regulation of phosphorylation level in the BR signaling pathway were significantly inhibited by 2.1 times and 2.8 times respectively, while the activities of *SMT*, *det* and *Dim*, the key genes of the BR synthesis pathway, were significantly inhibited and activated, respectively. The results were inconsistent with the view of Sun et al. that the regulation of rice stem elongation by *sui2* was unrelated to the genes involved in the BR synthetic pathway. This indicated that the sheathed spike gene not only affected hormone content but also regulated hormone signal transduction in the stems. In addition, there should be some level of balance between GA and BR signals, the detailed mechanism warrants more investigation.

WUSCHEL-related homeobox family genes play important and various roles in plant growth and development [26]. To date, just one WUSCHEL-related homeobox family gene, DWARF TILLER1 (*DWT1*) [27], was found to regulate the elongation of intercalary meristem of rice, which is homologous to the members of *WOX8* and *WOX9* in *Arabidopsis thaliana* and controls the developmental uniformity of the main shoot and tillers in rice. In *dwt1* mutant, the 2nd, 3rd and 4th internodes were significantly shortened, but the elongation of the uppermost internode almost was not affected, which has a great difference with the phenotype of *TaWUS*-*like*-OE. In our *TaWUS-like*-OE lines, the shortening of internodes occurred specifically in the uppermost and secondary internodes. Moreover, based on their amino acid sequences, the two genes belong to a different subfamily of the WUSCHEL-related homeobox family. *DWT1* belongs to the intermediate type, while *TaWUS-like* belongs to the WUSCHELE type, which is highly homologous to the *wox5*-*like* gene in rice and *Aegilops chinensis*, respectively. To our knowledge, this is the first key gene reported in wheat that has a function in regulating the elongation of the internode. Results indicated that different WUSCHEL family members may have different mechanisms in regulating stem development. In addition, both our results and those of Wang et al. found that these two genes were highly expressed in young spikes and developing grains but could not be detected in the internodes. Therefore, we suggest that the WUSCHEL family genes should have a key regulatory function or share a common mechanism, in which may transfer an unknown signal or small molecular substances from the grains to the internodes in a timely fashion thus significantly affecting the elongation of the plant stems. In the study of rice, Wang et al. believed that *DWT1* may affect cell division and elongation via regulating the genes expression of *cyclin* and *expansin*. Among the changes in the expression of downstream genes regulated by *TaWUS-like*, we also found that the activities of eight *cyclins* and eight *expansins* belong to EXPA/EXPB family were significantly inhibited. Wang et al. reported that the cyclins and their dependent protein kinases together are closely related to cell division and differentiation, while the *expansin* of the EXPA/EXPB family is highly associated with cell wall loosening and cell elongation. Therefore, the decreased activities of these genes could directly lead to the inhibition of cell division and insufficient elongation of longitudinal cells in the stem.

## 4. Materials and Methods

### 4.1. Plant Materials and Growth Conditions

The *TaWUS-like*-OE transgenic materials (homozygous T_8_ lines) were generated and provided by the Wheat Genetic Resources Group, the Chinese Academy of Agricultural Sciences. The wheat variety KN199 was selected as the receptor and the full-length cDNAs of *TaWUS-like* were inserted into pCAMBIA3301 vector and then transformed into wheat callus by using *Agrobacterium tumefaciens*-mediated transformation method in the Transgenic Center of the Chinese Academy of Agricultural Sciences. The detailed *Agrobacterium* transformation methods as described in Wang et al. [28]. The positive transgenic lines were double determined by the methods of PCR and the *Bar* strip test (QuickStix kit, Envirologix, Portland, ME, USA). The transcriptional levels and copy numbers of the target gene in these transgenic lines were detected respectively by Q-RT-PCR and genetic methods (PCR or Q-RT-PCR primers used were provided in Supplementary data 1). All transgenic lines were grown in the Shunyi transgenic experimental fields in Beijing with standard water and fertilizer management. Transgenic lines and WT plants were planted in four rows each, with 2 m of line length and 30 cm of row distance and 20 seeds per row. About 300 m away, another replication with the same planting arrangement was used. Samples were collected from the same block. Thirty OE homozygous plants (10 plants for each transgenic line, 3 independent transgenic lines) and WT plants were selected randomly to investigate the agronomic traits including 1000-grain weight, plant height, tillering number and stem length of the uppermost and secondary internode (Supplementary data 2).

### 4.2. Cytological Observation of the Stem Internodes and Flag Leaf

The flag leaf and uppermost internode of *TaWUS-like-*OE lines and WT plants were sampled at heading stage, and then paraffin-embedded and sliced according to the method of Ji et al. [29]. Briefly, the samples were cut into 1–2 cm, which were fixed at 4 °C at least 12 h in 50% (*v*/*v*) formalin acetic acid-alcohol solution, and samples were stained with 1% saffron and 0.5% solid green respectively for 2 h and 20 s. The conventional paraffin section process was used for tissue dehydration, saffron fixation, embedding and slicing, which were observed and photographed by stereomicroscope (ZEISS.V20, Jena, Germany). Whereafter, cell diameter and length were measured with straight lines in K-Viewer software (ver. 2.7.2.0, KFBIO Company, Ningbo, China). Each sample had 3 biological repeats. 

### 4.3. Determination of Hormone Level in Flag Leaf and Stem Internodes

The levels of GA and BR were determined from the uppermost and second internode and flag leaf of *TaWUS-like-*OE lines and WT plants at heading stage according to the method of Yi et al. [30]. A total of 100 mg samples were frozen and ground in liquid nitrogen, after that, 1 mL extraction solution was added (acetonitrile:water = 1:1) and kept on ice for 4 h. The supernatant was collected after centrifuging at 12,000× *g* rpm at 4 °C for 10 min. The sample was enriched by vacuum and 0.1 M ammonia solution was added to a final constant volume of 2 mL, and then added put the MAX column (The column was activated in advance with 4 mL methanol and 2 mL 0.1 M ammonia solution in turn). The column was rinsed with 2 mL 0.1 M ammonia solution then and 2 mL 0.1 M ammonia 60% methanol solution, and 0.2 mL methanol for dissolution was added. Afterward, the hormone level was measured using the ultra-high performance liquid chromatography-mass spectroscopy system (UHPLC-MS). The standards are purified 99% BR, GA_3_ and GA_4_ (Sigma-Aldrich, St. Louis, MO, USA). 

Chromatographic separation of the metabolites was performed on a Waters UHPLC system (Vanquish, Thermo, Waltham, MA, USA) equipped with a waters HSS T3 (50 × 2.1 mm, 1.8 μm Waters, Milford, MA, USA) column, with the injection volume of 2 μL and the column temperature of 40 °C. Mobile phase A is 0.1% acetic acid-acetonitrile and mobile phase B is 0.1% acetic acid-water. The mobile phase gradient is 0–1 min, 10% A; 1–3 min, 10% A-60% A; 3–8 min, 60% A; 8–8.1 min, 60% A-10% A and 8.1~9.0 min, 10% A. The mass spectrometric data were collected using a Thermo UHPLC-Q Exactive Mass Spectrometer equipped with an electrospray ionization (ESI) source operating in either positive or negative ion mode. The optimized mass spectrometry analysis conditions were as follows: sheath gas 40; auxiliary gas 10; ion spray voltage −2800 V; temperature 350 °C; the temperature of the ion transport tube is 320 °C. Each sample had 3 biological replicates.

### 4.4. RNA-Seq Analysis

Based on the method of Chi et al. [23], the total RNA was extracted from wheat grain at 10 days after anthesis of three *TaWUS-like-*OE lines and WT plants by using TIANGEN RNA kit (concentration ≥ 400–500 ng/μL). The quality and quantity of RNA samples were assessed by 1% RNase-free agarose gel electrophoresis and NanoDrop 2000 Spectrophotometer (Thermo), respectively. The qualified samples were sent to BaiXu Biotechnology Company for cDNA library construction and Illumina HiSeq™ 2500 sequencing (pair-end analysis, San Diego, CA, USA). After quality control and filtering, the clean reads were mapped to the Chinese Spring reference genome (IWGSCv1.0). The differentially expressed genes (DEG, Fold Change ≥ 2 and FDR < 0.01) were retained as fragments per kilo base per million reads (FPKM) and the levels of DEG were presented in the form of cluster heat map (Original data provided in Supplementary data 3), and analysis was performed by Edge R software (4.1, Brisbane, Australia). Each sample had 3 biological replicates. 

### 4.5. Statistical Analysis

One Way ANOVA method and turkey test was performed for variance and significance analysis with the SPSS (21.0, Chicago, IL, USA). All statistical experiments were performed no less than three times. The data were presented as the mean values ± standard deviation.

## 5. Conclusions

To our knowledge, *TaWUS-like* was the first and important sheathed spike gene in wheat reported to date. Overexpression of this gene results in a significant half- or full-wrapped spike enclosed by flag leaf sheaths with a shortened stem. The extremely shortened uppermost and secondary internodes of the stem and an insufficient elongation of the longitudinal length of cells in the internodes are the main reasons for the sheathed spike, which in turn are closely related to the significant decrease in GA_3_ content and the increase in BR content in internodes. GA and BR together are involved in the regulation of genes expression of stem development, and *TaWUS-like* may be the key factor to coordinate these two hormone pathways. *TaWUS-like* could regulate the synthesis and signal transduction of GA and/or BR and thus can affect the levels and functions of GA and BR in the plant stem. It also can influence the gene expression of *cyclin* and *expansin*, and in the end, caused stem shortening and plant dwarfing in wheat. This study has laid a solid foundation for further studies of the mechanism of GA- and BR-related genes controlling sheathed spikes in wheat.

## Data Availability

The detection of transgenic lines and primers used in identification and transcriptional level analysis were provided in Supplementary data 1; The phenotype data of transgenic lines and WT plants including TKW, plant height, tiller number and length of internode were provided in Supplementary data 2; The transcriptome expression data was provided in Supplementary data 3.

## Supplementary Materials

The following are available online at https://www.mdpi.com/article/10.3390/ijms222011210/s1.
