# Supplementary material for: A Sheathed Spike Gene, TaWUS-like Inhibits Stem Elongation in Common Wheat by Regulating Hormone Levels
Source: Int J Mol Sci. 2021 Oct 18;22(20):11210. doi: 10.3390/ijms222011210 (PMC8541442; doi:10.3390/ijms222011210)
Supplement: Supplementary file 1 [file ijms-22-11210-s001.zip › Supplementary data1.pdf]

Primers used in the determination of *TaWUS-like-OE* lines

TaW2WX-F: 5'-TTTAGCCCTGCCTTCATACG-3'

TaW2WX-R: 5'-GGAACCAGTAGAAGACGTTC-3'

Product size, 423 bp

Primers used in detection of transcriptional levels of *TaWUS-like* in transgenic OE lines

Q-TaW2-5D-F: 5'-CGCCGCCGCCACCGTCGC-3'

Q-TaW2-5D-R: 5'-GCCACCGCCTCCACTCTG-3'

Product size, 211 bp

The copy number determination of *TaWUS-like* in transgenic OE lines

We total have obtained 20 independent transgenic lines. Copy number was determined by using genetic methods. Based on the segregation ration of T_1 plant, we found 60 % of transgenic lines genome have the segregation ration of 3:1 (12 transgenic lines, single copy), the other 40 % lines have the segregation ration of 15:1 (8 transgenic lines, two copies). Afterward, we just select single-copy transgenic lines to evaluate the phenotype, after 6-7 generation self-pollination of transgenic lines to obtain the homozygous lines (single spike inheritance, no segregation any more). Here, in this manuscript, three independent transgenic lines are homozygous T8 lines.

The transcriptional levels detection of *TaWUS-like* in transgenic OE lines (partly show)

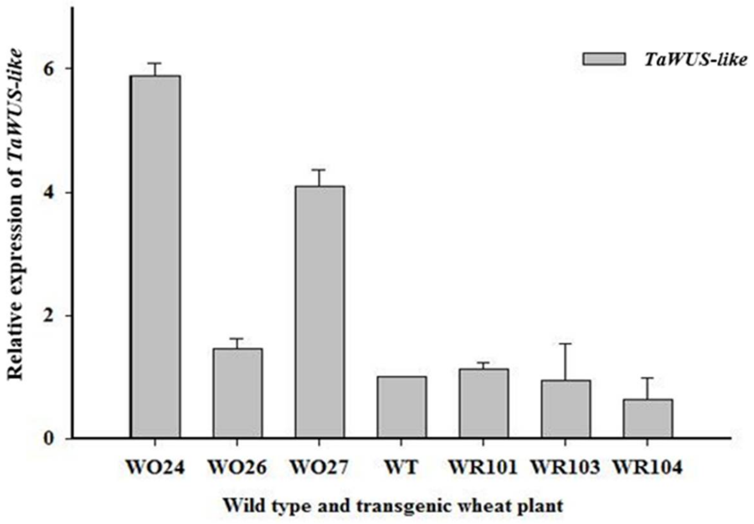

Figure S1 Transcriptional level analysis of target genes

WO, wus-like overexpression transgenic lines, WR, wus-like RNAi-interferencing transgenic lines WT, transgenic receptor kenong 199, which is a high-yield wheat variety (mean value of TKW is about 42 g) and easy to transform, and have a mature transformation protocol and system in CAAS. Note: In this manuscript, we selectWO24 and WO27 transgenic lines, the transcriptional level of WO57 is not provided here, its level is at least 3 times higher than that of the WT. The RNAi-interferencing lines didn't provided in this manuscript because the phenotype of sheathed spike is the same as that of WT.

The detection of transgenic lines by PCR (show partly results)

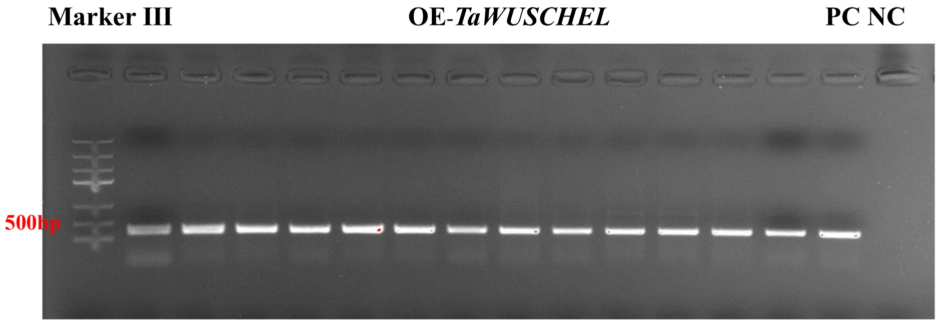

Figure S2 ***TaWUS-like-OE*** **transgenic lines detection**

PC, transgenic positive control, plasmids control; **NC**, transgenic Negative control, mix control. F primer, located in the vector sequence of UBI promoter (Maize); R primer, located in the CDS sequence of target gene (the length of the product here is about 423 bp).

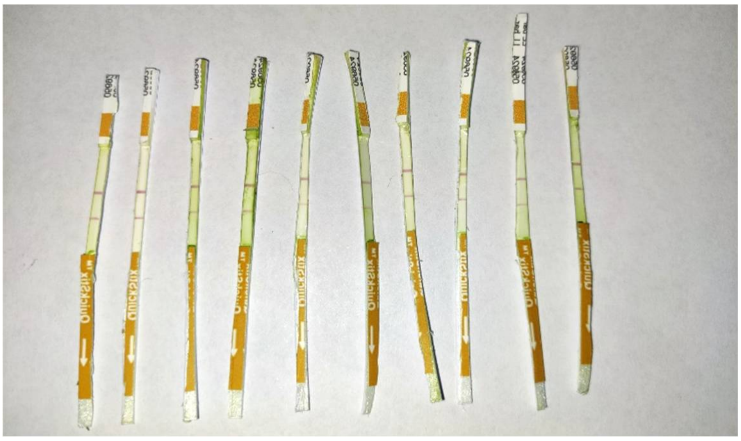

Figure S3 Bar stripe detection

Double strip show positive transgene, Single strip show negative transgene
